# Ring-alkyl connecting group effect on mesogenic properties of *p*-carborane derivatives and their hydrocarbon analogues

**DOI:** 10.3762/bjoc.5.83

**Published:** 2009-12-30

**Authors:** Aleksandra Jankowiak, Piotr Kaszynski, William R Tilford, Kiminori Ohta, Adam Januszko, Takashi Nagamine, Yasuyuki Endo

**Affiliations:** 1 Organic Materials Research Group, Department of Chemistry, Vanderbilt University, Box 1822 Station B, Nashville, TN 37235, USA, Phone/Fax: (615) 322-3458; 2 Tohoku Pharmaceutical University, 4-4-1, Komatsushima, Aoba-ku, Sendai 981-8558, Japan

**Keywords:** *p*-carborane, liquid crystals, structure-property relationship

## Abstract

The effect of the phenyl–alkyl connecting group on mesogenic properties of several series of isostructural compounds containing *p*-carborane (**A** and **B**), bicyclo[2.2.2]octane (**C**), and benzene (**D**) was investigated using thermal and optical methods. Results demonstrated that mesophase stability in the series containing **A**–**D** follows the order (Alk)CH_2_CH_2_– < (Alk)OOC– < (Alk)CH_2_O– < (Alk)COO–. Surprisingly, the connecting groups (Alk)CH_2_CH_2_– and (Alk)OOC– destabilize the mesophase significantly stronger for carboranes (**A** and **B**) than for carbocyclic derivatives (**C** and **D**). Analysis indicates that this effect may have quadrupolar and conformational origin.

## Introduction

**Figure 1:**
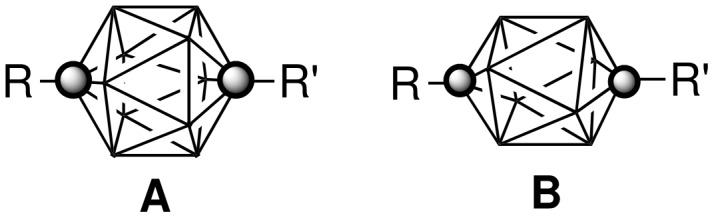
The molecular structures of 1,12-dicarba-*closo*-dodecaborane (12-vertex *p*-carborane, **A**) and 1,10-dicarba-*closo*-decaborane (10-vertex *p*-carborane, **B**). Each vertex corresponds to a BH fragment and the sphere represents a carbon atom.

During the past decade, we have been investigating mesogenic derivatives of *p*-carboranes **A** and **B** (Figure 1) in the context of fundamental and applied studies of liquid crystals and development of new materials for electrooptical applications [1–23]. *p*-Carboranes belong to an extensive family of *closo*-boranes and are characterized by 3-dimensional σ-aromaticity and high-order symmetry axis [23]. Therefore, it is of interest to understand how the electronic properties of the two clusters and their unusual molecular symmetry and size affect bulk properties of mesogens. Through extensive comparison of isostructural mesogenic derivatives of *p*-carboranes (**A** and **B**), bicyclo[2.2.2]octane (**C**), and benzene (**D**), we have been probing fundamental aspects of structure-property relationships in liquid crystals such as the effect of conformational properties [1–2], the structure of the linking group [5], and tail fluorination [18–19] on mesophase stability, and also the effectiveness of shielding of a lateral substituent [8,16,20] and chirality transfer phenomena [17]. Results of these studies are important for the design of new materials and optimizing of their properties for applications.

**Figure 2:**
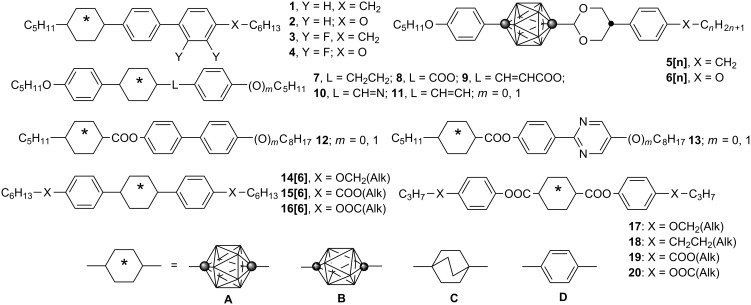
The molecular structures of derivatives **1**–**20**.

During our investigation of structurally related series of mesogens containing rings **A**–**D** (Figure 2), it became apparent that the benzene ring–alkyl chain connection has a distinctly different impact on phase stability in derivatives of *p*-carborane (**A**) than in their isostructural carbocycles. For instance, a larger stabilization of the nematic phase, upon CH_2_→O replacement, was observed in *p*-carborane mesogens relative to benzene analogues. Thus, in series **1**–**4**, the nematic phase is stabilized by about 14 K more for the pairs **1A**/**2A** and **3A**/**4A**, than for terphenyl (**D**) and bicyclo[2.2.2]octane (**C**) analogues (Figure 2). Similarly high values for phase stabilization of about 34 K are observed in pairs of alkyl and alkoxy dioxane derivatives **5[n]** and **6[n]** [4], as compared to similar benzene mesogens [24]. Also in series **7**–**11** the introduction of the connecting oxygen atom gives a larger increase in mesophase stability by an average of 6 ± 2 K for the 12-vertex *p*-carborane derivatives than for their benzene analogues [5]. However, in series **12** and **13** the effect of incorporation of the O atom as the connecting group is practically the same for all ring systems [15].

A recently developed series of isostructural mesogens allows to analyze the effect of the replacement of an alkoxy in **14[6]** with an ester group in **15[6]**. The CH_2_O→OOC exchange dramatically destabilized the nematic phase for the 10- and 12-vertex *p*-carborane derivatives, while a much smaller effect was observed for the carbocycles [25]. Series **14** and **15** [25] and also diesters **17** [2] provide an opportunity for further investigation of this interesting phenomenon. Therefore, we focused on series **14**–**16** to investigate the CH_2_O, COO, OOC connecting groups, and also on series **17**–**20** to study the CH_2_O, CH_2_CH_2_, COO, OOC groups.

Here we describe the preparation of an isostructural series of diesters **16[6]** and **18B**, and also tetraesters **19** and **20**, and a detailed comparative analysis focusing on the impact of the connecting group on mesogenic properties. The analysis is aided by molecular modeling of the pertinent molecular fragments. In addition, we report two homologues of **16A[6]**, diesters **16A[5]** and **16A[7]**.

## Results

### Synthesis

**Scheme 1:**
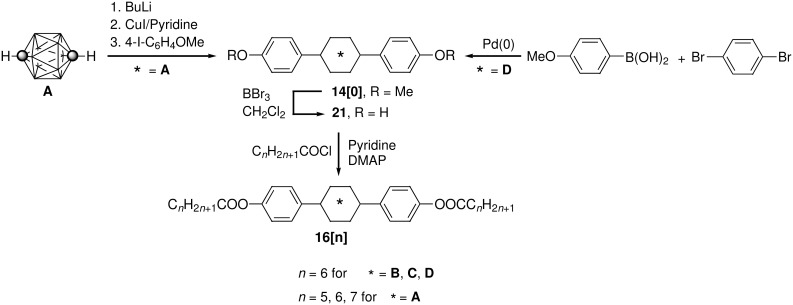
Preparation of diesters **16[n]**.

Diesters **16[n]** were prepared from diphenols **21** and appropriate carboxylic acid chlorides in the presence of a base as shown in Scheme 1. The requisite diphenols **21** were obtained in nearly quantitative yields by treating the corresponding dimethoxy derivatives **14[0]** with BBr_3_. This procedure represents a significant improvement to the original preparation of 1,12-bis(4-hydroxyphenyl)-*p*-carborane (**21A**) from **14A[0]** [26] and 1,4-bis(4-hydroxyphenyl)benzene (**21D**) [27]. The preparation of diphenol **21C** will be described elsewhere [25]. The dimethoxy carborane derivative **14A[0]** was obtained using the Wade’s arylation procedure [28] of *p*-carborane (**A**) with 4-iodoanisole as described before [26]. The 10-vertex analogue **14B[0]** was prepared in a similar way and will be described elsewhere [25]. 4,4”-Dimethoxyterphenyl **14D[0]** was prepared in 84% yield from 1,4-dibromobenzene and (4-methoxyphenyl)boronic acid using the Suzuki coupling procedure [29]. This method is comparable to other Pd(0)-assisted methods for the synthesis of **14D[0]** [30–32].

**Scheme 2:**
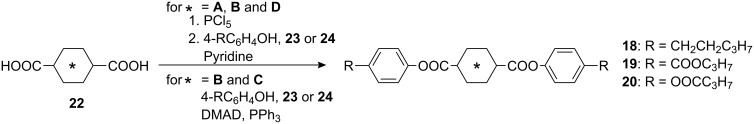
Preparation of esters **18**–**20**.

The 10-vertex *p*-carborane diester **18B** was obtained from the corresponding dicarboxylic acid **22B** [33] and 4-pentylphenol (Scheme 2) according to a recently described procedure [2]. The two series of tetraesters **19** and **20** were prepared from the appropriate dicarboxylic acid **22** and phenols **23** and **24**, respectively. *p*-Carborane-1,12-dicarboxylic acid **22A** and terephthalic acid (**22D**) were converted to the corresponding acid chlorides using PCl_5_ and then reacted with phenols in the presence of a base. The previously described method [2] for the preparation of esters of bicyclo[2.2.2]octane-1,4-dicarboxylic acid (**22C**) was unsuccessful and the desired esters **19C** and **20C** were obtained from the diacid and appropriate phenol using the classical Mitsunobu procedure [34]. A similar procedure was used for the preparation of tetraester **20B**, while **19B** was prepared more efficiently using the acid chloride method. Ester **19D** has been reported in the literature [35].

**Scheme 3:**
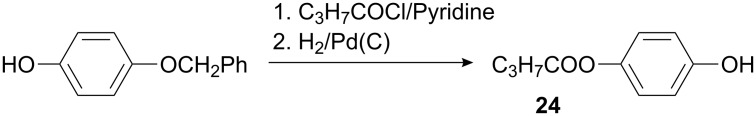
Preparation of phenol **24**.

Phenol **24** was prepared by acylation of 4-benzyloxyphenol with butyryl chloride followed by removal of the protective benzyl group under reductive conditions as described in the literature [36] (Scheme 3).

### Mesogenic properties

Transition temperatures and enthalpies of the newly prepared compounds were determined by differential scanning calorimetry (DSC). The phase types were assigned by comparison of microscopic textures observed in polarized light with those published for reference compounds [37–39]. Results for these and also their structural analogues are shown in Table 1 and Table 2.

**Table 1:** Transition temperatures (°C) for selected liquid crystals.*^a^*

	*	**A**	**B**	**C**	**D**
		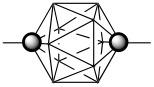	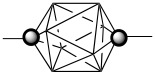	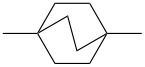	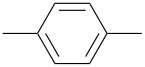

	**X**	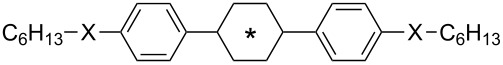			
**14[6]**	–CH_2_O–(Ph)	Cr 96 N 98 I*^b^*	Cr 73 N 105 I*^b^*	Cr 98 SmB 161 SmA 179 I*^b^*	Cr*^c^* 182 SmF 218 SmI 219 SmC 232 SmA 235 I*^b^*
**15[6]**	–OOC–(Ph)	Cr 112 (N 31) I*^b^*	Cr 65 (N 11) I*^b^*	Cr*^d^* 114 SmA 148 I*^b^*	Cr 134 SmC 143 SmA 183 I*^b^*
**16[6]**	–COO–(Ph)	Cr 108 N 132 I	Cr*^e^* 102 N 136 I	Cr*^f^* 102 X 205 N 207 I	Cr 66 X 96 SmF 226 SmI 232 SmC*^g^* 250 SmA 251 I
		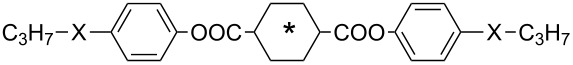			
**17**	–CH_2_O–(Ph)	Cr 137 N 182.6 I*^h^*	Cr*^i^* 111 N 183.4 I*^h^*	Cr 112 N 229.5 I*^h^*	Cr 189 N 235 I*^j^*
**18**	–CH_2_CH_2_–(Ph)	Cr 106 N 118 I*^k^*	Cr 85 N 110 I	Cr 98 N 173 I*^l^*	Cr 155 N 181 I*^m^*
**19**	–OOC–(Ph)	Cr 203 (N 139) I*^g^*	Cr 160 (N 128) I	Cr 121 N 195 I	Cr 130 SmA 207 N 221 I*^n^*
**20**	–COO–(Ph)	Cr 133 N 230 I	Cr 120 N 234 I	Cr 133 N 275 I	Cr 230 N 287 I

*^a^*Obtained on heating; Cr: crystal, Sm: smectic, N: nematic, I: isotropic, X: unidentified phase. Transition enthalpies for new compounds are listed in the SI. *^b^*Ref. [25]. *^c^*Cr – Cr transition at 108 °C. *^d^*Cr – Cr transition at 100 °C. *^e^*Cr – Cr transition at 73 °C (14.9 kJ/mol). *^f^*Cr – Cr transition at 33 °C (11.6 kJ/mol). *^g^*Optical determination obtained on cooling .*^h^*Ref. [2]. *^i^*Cr – Cr transition at 70 °C. *^j^*Ref. [40]. *^k^*Previously reported Cr 104 N 114 I, ref. [21]. *^l^*Ref. [41]. *^m^*Ref. [42]. *^n^*Ref. [35].

**Table 2:** Transition temperatures (°C) for **16A[n]**.*^a^*

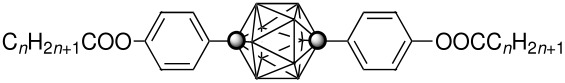	
**n**	Transition temperatures

5	Cr_1_ 66 Cr_2_ 120 N 155 I
6	Cr 108 N 132 I
7	Cr_1_ 76 Cr_2_ 92 N 124 I

^a^Obtained on heating; Cr: crystal, N: nematic, I: isotropic. Transition enthalpies are listed in the SI.

**Figure 3:**
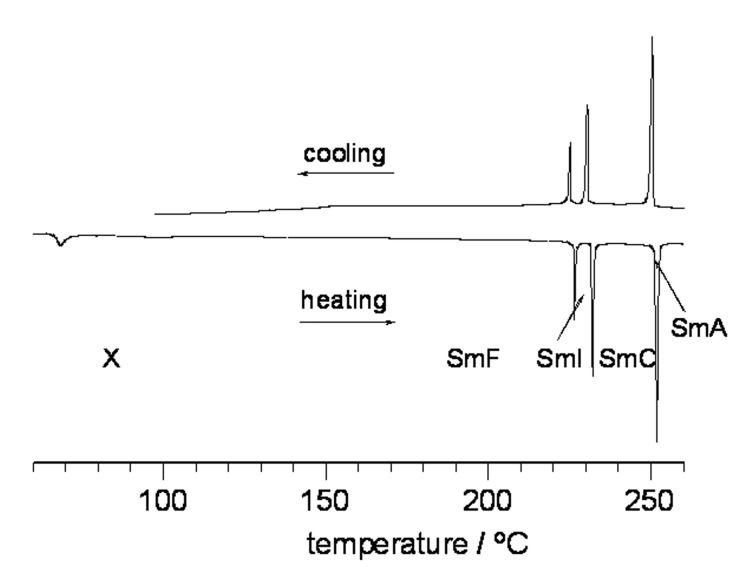
Partial DSC trace for **16D[6]**. Heating rate 5 K/min.

**Figure 4:**
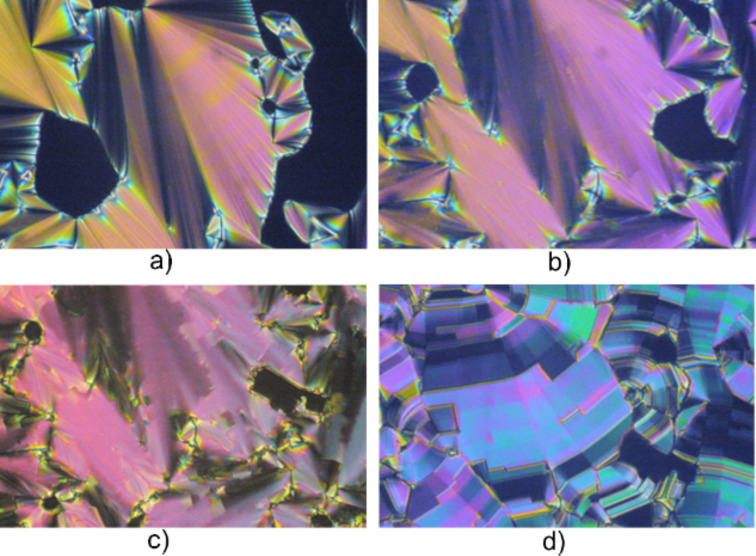
Optical textures of **16D[6]** obtained for the same region of the sample upon cooling: (a) SmA growing from isotropic (251 °C), (b) focal-conic texture of SmC (242 °C), (c) SmI (229 °C), and (d) broken focal-conic texture of SmF (211 °C). Magnification × 60.

All *p*-carborane derivatives in series **14**–**20** exhibit exclusively the nematic phase. Similar nematic behavior is observed for carbocycles in series **17**–**20** with the exception of **19D**, which exhibits a SmA phase in addition to a N phase. In contrast, most carbocyclic derivatives in series **14[6]**–**16[6]** display only smectic and soft crystalline polymorphs. The bicyclo[2.2.2]octane derivative **16C[6]** is the only exception and exhibits a narrow range nematic phase above a soft crystalline phase designated as L or E on the basis of viscosity, ability to supercool, and optical textures. In general, bicyclo[2.2.2]octane derivatives **14C[6]**–**16C[6]** exhibit orthogonal phases (SmA and SmB), while the terphenyl analogues display a rich smectic polymorphism involving mainly tilted phases. The terphenyl derivatives **14D[6]** and **16D[6]** exhibit the most interesting polymorphism in the series with 4 smectic phases and possibly a soft crystalline modification such as a G phase below the SmF phase in the latter. A DSC trace for **16D[6]** is shown in Figure 3, and representative textures of its mesophases are presented in Figure 4. The tilted phases in both terphenyl compounds were identified by the appearance and subsequent characteristic changes of the Schlieren textures in the homeotropic regions of the SmA phase upon cooling.

In general, the order of phase stability for all five series follows **A** ~ **B** < **C** < **D**. Derivatives of both *p*-carboranes **A** and **B** exhibit similar stability of the nematic phase, with the exception of **15[6]** and **19** for which the monotropic nematic phase of the 10-vertex carborane derivatives is significantly less stable (<20 K) than that of the 12-vertex analogues.

Analysis of three homologues **16A[n]** demonstrated the decreasing stability of the nematic phase with increasing chain length from *T*_NI_ of 155 °C for *n* = 5 to 124 °C for *n* = 7 (Table 2). Investigation of the 4,4″-dimethoxyterphenyl **14D[0]** revealed a high temperature nematic phase (Cr 277 N 295 I), which is in disagreement with the original literature report [43].

### Comparative Analysis

**Figure 5:**
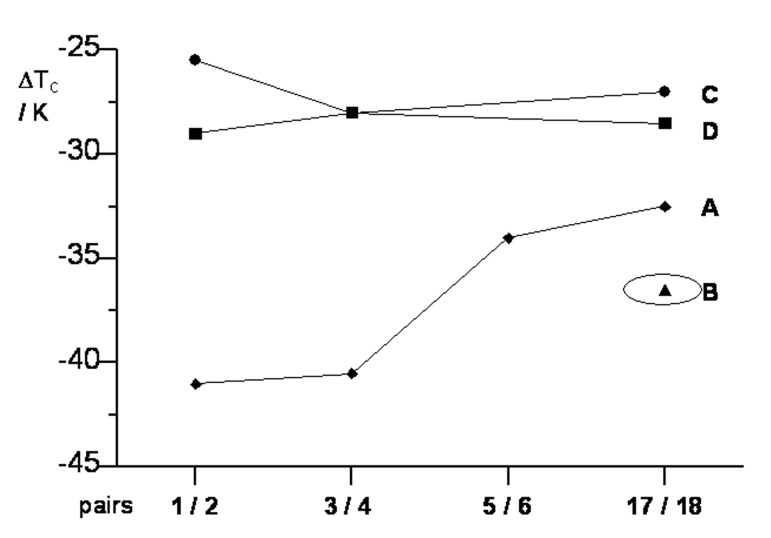
The change in the clearing temperature Δ*T*_c_ upon substitution –OCH_2_– → –CH_2_CH_2_– in selected pairs of compounds. The lines are guides for the eye.

**Figure 6:**
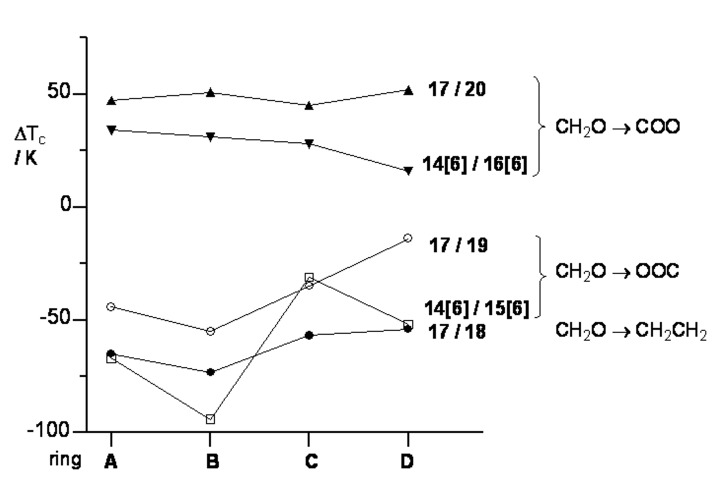
The change in the clearing temperature Δ*T*_c_ upon replacing of the –OCH_2_– connecting group with another in selected pairs of compounds. The lines are guides for the eye.

Mesogenic properties of structurally analogous pairs were compared, and the results are presented in Figure 5 and Figure 6.

#### The –OCH_2_– → –CH_2_CH_2_– substitution

A comparison of *T*_NI_ for compounds in series **17** [2] versus their isostructural analogues **18**, in which the linking oxygen atom is replaced with –CH_2_–, demonstrates that the presence of the oxygen atom increases the phase stability by about 55 K (or 27 K per alkoxyphenyl group) for the carbocyclic compounds. In contrast, the difference in *T*_NI_ is larger by 10 K for *p*-carborane **A** and 18 K for *p*-carborane **B** (Figure 5 and Figure 6). These results are consistent with earlier findings for pairs **1**/**2**, **3**/**4**, and **5**/**6** (Figure 5) in which a particularly large impact of the –OCH_2_– → –CH_2_CH_2_– substitution on *T*_NI_ is observed for the rigid biphenyl derivatives **1**–**4**.

#### The –OCH_2_– → –OOC– and –OCH_2_– →–COO– substitution

Data in Table 1 demonstrate that the replacement of the heptyloxy group with heptanoyloxy in **14[16]**/**16[6]** and butoxy with butanoyloxy in **17**/**20** results in an increase of the *T*_NI_ by about 30 K and 45 K, respectively, for all structural units **A**–**D**. The only exception is the pair **14D[6]**/**16D[6]** for which the change in *T*_NI_ is only 16 K. The larger change of *T*_NI_ for pairs **17**/**20** than for **14[6]**/**16[6]** is consistent with attenuation of the substitution effect by the shorter alkyl chain in the latter (–C_3_H_7_ vs –C_6_H_13_).

In contrast, replacement of the oxymethylene linking group with a carboxy group of reversed orientation relative to the core (pairs **14[6]**/**15[6]** and **17**/**19**) leads to significant destabilization of the mesophase, and the magnitude of the effect markedly depends on the nature of the central structural element (Figure 6). Thus, for derivatives of carbocycles **C** and **D**, *T*_c_ decreases less than 55 K for **14[6]**/**15[6]** and less than 40 K for **17**/**19**, while for the *p*-carboranes the decrease is larger, reaching a value of 94 K for the pair **14B[6]**/**15B[6]**.

Data in Table 1 also allow for assessment of the impact of the orientation of the connecting carboxyl group on *T*_NI_ as a function of the central structural element. Thus, in pairs **16[6]**/**15[6]** and **20**/**19**, the change of carbonyloxy to oxycarbonyl leads to a marked phase destabilization for all structural elements **A**–**D**. Consistent with our previous analysis, the effect is much stronger for *p*-carboranes (>90 K) than for carbocycles (<80 K) with the typical order: **D**, **C** < **A** < **B**.

### Molecular Modeling

**Figure 7:**
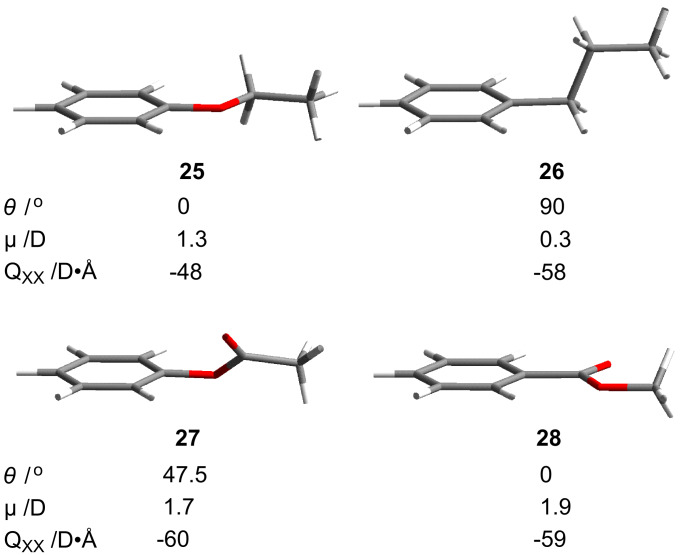
Equilibrium ground state geometries (B3LYP/6-31G(d)) for benzene derivatives: ethoxybenzene (**25**), propylbenzene (**26**), phenyl acetate (**27**), and methyl benzoate (**28**) and pertinent molecular parameters: dihedral angle θ, dipole moment μ, and quadrupole moment tensor Q_XX_ perpendicular to the ring plane.

For a better understanding of the terminal substituent’s impact on the conformational ground state of the molecules, four benzene derivative models, **25**–**28**, were optimized at the B3LYP/6-31G(d) level of theory, and their equilibrium geometries are presented in Figure 7. Results show that the replacement of the oxygen atom with a –CH_2_– group reorients the terminal chain from co-planar, in the conformational ground state of ethoxybenzene (**25**), to the orthogonal position relative to the benzene ring plane in propylbenzene (**26**). Replacement of the –CH_2_O– fragment with the –COO– group leads to an increase of angle θ between the ring and substituent planes to about 50° in phenyl acetate (**27**). Reversing the connectivity of the ester group (–COO– → –OOC–) results in return to the co-planar orientation of the substituent in benzoate **28**. The computational results are consistent with experimental findings for anisole [44] and ethylbenzene [45], and solid-state structures for compounds containing fragments **25**–**28** [46].

Overall, the interplanar angle θ between the ring and substituent increases in the series **25**, **28** < **27** < **26** or –OR, –C(O)OR < –OOCR < –CH_2_R.

Further analysis of the computational results demonstrates that molecular dipole moment μ increases in the following order: **26** < **25** < **27** < **28**. The quadrupole moment tensor Q_xx_ perpendicular to the benzene ring is larger for the esters than for alkyl or alkoxy derivatives.

## Discussion and Conclusion

Results presented in Table 1 are in agreement with general trends [47–48] and demonstrate that the replacement of –OCH_2_– with –OOC– increases *T*_NI_, while replacement with –CH_2_CH_2_– or –COO– decreases *T*_NI_. Overall, the effectiveness of the connecting group in mesophase stabilization follows the order: –OOC(Alk) > –OCH_2_(Alk) > –COO(Alk) > –CH_2_CH_2_(Alk). The magnitude of the effect for the –OCH_2_– → –OOC– replacement is practically independent of the central structural element **A**–**D**. In contrast, the decrease in *T*_NI_ upon substitution of –OCH_2_– with –CH_2_CH_2_– or –COO– is stronger for *p*-carborane derivatives (**A** and **B**) than for their carbocyclic analogues. This effect is observed for compounds in which the *p*-carborane is connected directly to the substituted benzene ring (**1**–**4**, **14**–**16**) or through a spacer (**5**, **6**, **17**–**20**).

The origin of the observed relative effectiveness of the connecting groups (–OOC(Alk) > –OCH_2_(Alk) > –COO(Alk) > –CH_2_CH_2_(Alk)) is unclear at the moment. In general, the phase stability is related to packing fraction, and for more compact anisometric molecules (high packing fraction) the clearing temperature is higher [49]. Thus, it can be expected that compounds with substituents preferring coplanar orientation with the aryl ring (–OR and –COOR) would exhibit higher mesophase stability than those with non-coplanar orientation (–CH_2_R and –OOCR). While this simple steric argument is consistent with data for pairs –CH_2_R/–OR, the effect of orientation of the carboxy group on mesophase stability is opposite. Therefore, steric arguments alone cannot explain the observed trend for these analogues.

Electronic effects also cannot sufficiently explain the observed trend in mesophase stability and the poor performance of the carboxyl group. Thus, the observed trend is inconsistent with the order of dipole moments calculated for the relevant molecular fragments **25**–**28** (Figure 7). According to the computational results, esters **27** and **28** have similar, and also the largest molecular dipole and quadrupole moments. Therefore, compounds containing these fragments would be expected to exhibit both similar and high mesophase stability. The data show otherwise and a large difference in *T*_C_ is observed for the isomeric esters (e.g. for **19B**/**20B** Δ*T*_NI_ = 80 K; for other examples see LiqCryst database [50]).

The origin of the observed excessive mesophase destabilization in *p*-carborane derivatives by the –COO(Alk) and –CH_2_CH_2_(Alk) substituents is even more puzzling. Data in Table 1 show that mesophase of *p*-carborane derivatives containing electron rich benzene rings (with the –OR and –OOCR substituents) is excessively stabilized relative to those containing either weakly donating (–CH_2_CH_2_R) or electron-withdrawing (–COOR) substituents. This suggests that intermolecular quadrupolar interactions between *p*-carborane and benzene ring may be responsible for the observed phase stabilization. Support for this hypothesis is provided by the finding that the connecting group affects bulk properties whether *p*-carborane is connected to the benzene ring directly or through a spacer. The observed larger effect of the –CH_2_CH_2_– → –OCH_2_– replacement in pairs **1A**/**2A** and **3A**/**4A** as compared to **17A**/**18A** suggests a role for the molecular dipole moment in phase stabilization. Since *p*-carboranes are moderately electron withdrawing substituents, the alkoxy derivatives have a larger dipole moment than the alkyl derivatives [16]. Alternatively, the effect can be due to higher rigidity of **1**-**4**, which attenuates the effect as compared to the more conformationally flexible diesters.

Overall, the analysis cannot distinguish one particular factor responsible for the impact of structural elements (**A**–**D**) on phase stabilization. Instead, a combination of conformational properties of structural elements **A**–**D** and substituents, their relative sizes [51], and electronic properties of the benzene ring bearing the substituent dictate mesogenic properties.

The present report concentrates on the systematic variation of the connecting group between the alkyl and phenyl ring, and its effect on phase stability. For completeness, we also mention one example of variation of the carborane–alkyl connecting group, and its impact on *T*_c_. Thus, a replacement of –CH_2_CH_2_– → –C≡C– destabilized the *T*_NI_ by over 150 K in bi-carborane derivatives, while a similar transformation in the biphenyl analogue leads to an increase of the clearing temperature [1,7]. This dramatic effect has been attributed to conformational properties of molecules in the condensed phase.

A more complete understanding of the impact of structural modification on bulk properties will emerge through further research on structure-property relationships and studying of other examples of structurally similar mesogens containing the four ring systems **A**–**D**.

## Experimental

Optical microscopy and phase identification were performed using a PZO “Biolar” polarized microscope equipped with a HCS400 Instec hot stage. Thermal analysis was obtained using a TA Instruments 2920 DSC. Transition temperatures (onset) and enthalpies were obtained using small samples (1–2 mg) and a heating rate of 5 K min^−1^ under a flow of nitrogen gas. For DSC and microscopic analyses, each compound was additionally purified by dissolving in CH_2_Cl_2_, filtering to remove particles, evaporating and recrystallization typically from hexanes or toluene/heptane mixture. The resulting crystals were dried in vacuum overnight at ambient temperature. 10- and 12-vertex *p*-carboranes (**A** and **B**) were purchased from Katchem s. r. o. (Prague, Czech Republic).

## Supporting Information

Synthetic procedures for compounds **14D[0]**, **16[n]**, **18B**, **19**, **20**, **24**, and analytical details are provided.

File 1 General methods and synthetic procedures
