# Supplementary material for: Ring-alkyl connecting group effect on mesogenic properties of p-carborane derivatives and their hydrocarbon analogues
Source: Beilstein J Org Chem. 2009 Dec 30;5:83. doi: 10.3762/bjoc.5.83 (PMC2839553; doi:10.3762/bjoc.5.83)
Supplement: File 1 — General methods and synthetic procedures [file Beilstein_J_Org_Chem-05-83-s001.pdf]

General methods and synthetic procedures

Supporting Information for

Ring-alkyl connecting group effect on mesogenic properties of *p*-carborane derivatives and their hydrocarbon analogues

Aleksandra Jankowiak[1], Piotr Kaszynski[1]*, William R. Tilford[1], Kiminori Ohta[2], Adam Januszko[1], Takashi Nagamine[2] and Yasuyuki Endo[2]

Address: [1]Organic Materials Research Group, Department of Chemistry, Vanderbilt University, Box 1822 Station B, Nashville, TN 37235, USA and [2]Tohoku Pharmaceutical University, 4-4-1, Komatsushima, Aoba-ku, Sendai 981-8558, Japan

Email: Piotr Kaszynski - piotr.kaszynski@vanderbilt.edu
Phone/Fax: (615) 322-3458

* Corresponding author

Table of Contents

1. General Methods p 2
2. Synthetic Procedures p 2
3. Thermal Analysis p 10
4. References p 12

1. General methods

^{1}H NMR spectra were obtained at the 270 or 300, 400 or 500 MHz field in $CDCl_3$ and referenced to TMS unless stated otherwise.

2. Synthetic procedures

1,4-Bis(4-methoxyphenyl)benzene (**14D[0]**) [1].

A mixture of (4-methoxyphenyl)boronic acid (1.52 g, 10 mmol), 1,4-dibromobenzene (1.18 g, 5 mmol) and $Pd(PPh_3)_4$ (116 mg, 0.1 mmol) in 1 M aq solution of Na_2CO_3 (20 mL, 20 mmol) and toluene (40 mL) was refluxed for 24 h under Ar atmosphere. The mixture was poured into water and AcOEt was added. Insoluble material was collected to give 1.22 g (84% yield) of terphenyl **14D[0]** as a colorless solid. Colorless prisms were obtained by recrystallization from xylene followed by vacuum sublimation: mp 279 °C (lit. [1] mp 273–274 °C); ^{1}H NMR (300 MHz) δ 3.86 (s, 6H), 7.00 (d, *J* = 8.8 Hz, 4H), 7.58 (d, *J* = 8.8 Hz, 4H), 7.61 (s, 4H); MS (EI), *m/z* 290 (M^+, 100%). Anal. Calcd for $C_{20}H_{18}O_2$: C, 82.73; H, 6.25. Found: C, 82.87; H, 6.23.

Diesters **16[n]**. General procedure.

To a solution of diphenol **21** (0.3 mmol) in of dry toluene or CH_2Cl_2 (5 mL) was added appropriate acid chloride (0.7 mmol), followed by pyridine (1 mL) and a catalytic amount of DMAP at room temperature. After stirring for 12 h, the mixture was poured into 2 N HCl and organic products were extracted with AcOEt. The organic layer was washed with sat. $NaHCO_3$ and brine, dried ($MgSO_4$) and then concentrated. The resulting crude diesters were purified by

column chromatography (silica gel, AcOEt/hexane, 1/10 or CH_2Cl_2/hexane, 1:1) and repeatedly recrystallized typically from AcOEt/hexane or isooctane/toluene mixture.

1,12-Bis[4-(hexanoyloxy)phenyl]-1,12-dicarba-*closo*-dodecaborane (**16A[5]**).

Colorless needles (AcOEt/*n*-hexane and then hexane): ^{1}H NMR (270 MHz) δ 0.92 (t, *J* = 7.0 Hz, 6H), 1.30–1.45 (m, 8H) 1.5–4.0 (brm, 10H), 1.73 (quint, *J* = 7.6 Hz, 4H), 2.52 (t, *J* = 7.6 Hz, 4H), 6.90 (d, *J* = 8.7 Hz, 4H), 7.23 (d, *J* = 8.7 Hz, 4H). Anal. Calcd for $C_{26}H_{40}B_{10}O_4$: C, 59.52; H, 7.68. Found: C, 59.22; H, 7.92.

1,12-Bis[4-(heptanoyloxy)phenyl]-1,12-dicarba-*closo*-dodecaborane (**16A[6]**).

Colorless needles (AcOEt/*n*-hexane): ^{1}H NMR (270 MHz) δ 0.90 (t, *J* = 6.8 Hz, 6H), 1.25–1.50 (m, 12H), 1.5–4.0 (brm, 10H), 1.72 (quint, *J* = 7.3 Hz, 4H), 2.52 (t, *J* = 7.2 Hz, 4H), 6.90 (d, *J* = 8.6 Hz, 4H), 7.23 (d, *J* = 8.7 Hz, 4H); HRMS, calcd. for $C_{28}H_{44}B_{10}O_4$: 552.4243. Found: 552.4217. Anal. Calcd for $C_{28}H_{44}B_{10}O_4$: C, 60.84; H, 8.02. Found: C, 60.88; H, 7.98.

1,12-Bis[4-(octanoyloxy)phenyl]-1,12-dicarba-*closo*-dodecaborane (**16A[7]**).

Colorless needles (AcOEt/*n*-hexane and then pentane): ^{1}H NMR (270 MHz) δ 0.89 (t, *J* = 6.9 Hz, 6H), 1.20–1.50 (m, 16H), 1.5–4.0 (brm, 10H), 1.72 (quint, *J* = 7.6 Hz, 4H), 2.52 (t, *J* = 7.7 Hz, 4H), 6.90 (d, *J* = 8.7 Hz, 4H), 7.23 (d, *J* = 8.7 Hz, 4H). Anal. Calcd for $C_{30}H_{48}B_{10}O_4$: C, 62.04; H, 8.33. Found: C, 61.83; H, 8.52.

1,10-Bis[4-(heptanoyloxy)phenyl]-1,10-dicarba-*closo*-decaborane (**16B[6]**).

The diester was obtained from crude diol **21B** prepared from the corresponding dimethoxy derivative [2] **14B[0]** as described for the 12-vertex analogue. The diester was purified by chromatography (silica gel, hexanes/CH_2Cl_2, 4:1) followed by repeated recrystallization (isooctane): ^{1}H NMR (300 MHz) δ 0.92 (t, *J* = 6.9 Hz, 6H), 1.33–1.50 (m, 12H), 1.5–4.0 (brm, 8H), 1.79 (quint, *J* = 7.5 Hz, 4H), 2.61 (t, *J* = 7.5 Hz, 4H), 7.17 (d, *J* = 8.7 Hz, 4H), 7.82 (d, *J* = 8.7 Hz, 4H). Anal. Calcd for $C_{28}H_{42}B_8O_4$: C, 63.56; H, 8.00. Found: C, 63.52; H, 8.03.

1,4-Bis[4-(heptanoyloxy)phenyl]bicyclo[2.2.2]octane (**16C[6]**).

The diester was obtained from diphenol **21C** [2] and purified by chromatography (silica gel, hexanes/CH_2Cl_2, 4:1) followed by double recrystallization (isooctane): ^{1}H NMR (300 MHz) δ 0.91 (t, *J* = 6.9 Hz, 6H), 1.30–1.47 (m, 12H), 1.75 (quint, *J* = 7.5 Hz, 4H), 1.95 (s, 12H), 2.55 (t, *J* = 7.5 Hz, 4H), 7.01 (d, *J* = 8.7 Hz, 4H), 7.36 (d, *J* = 8.7 Hz, 4H). Anal. Calcd for $C_{34}H_{46}O_4$: C, 78.72; H, 8.94. Found: C, 78.76; H, 8.99.

1,4-Bis[4-(heptanoyloxy)phenyl]benzene (**16D[6]**).

The diester obtained from diphenol **21D** was purified by chromatography (silica gel, hexane/CH_2Cl_2, 3:1) followed by recrystallization (AcOEt/CH_2Cl_2) to give colorless leaflets: ^{1}H NMR (300 MHz) δ 0.92 (t, *J* = 6.9 Hz, 6H), 1.25–1.51 (m, 12H), 1.78 (quint, *J* = 7.4 Hz, 4H), 2.59 (t, *J* = 7.5 Hz, 4H), 7.17 (d, *J* = 8.8 Hz, 4H), 7.62 (d, *J* = 8.7 Hz, 4H), 7.64 (s, 4H); MS (EI), *m/z* 486 (M^+), 262 (100%). Anal. Calcd for $C_{32}H_{38}O_4$: C, 78.98; H, 7.87. Found: C, 79.17; H, 7.93.

Bis(4-pentylphenyl) 1,10-dicarba-*closo*-decaborane-1,10-dicarboxylate (**18B**).

A suspension of *p*-carborane-1,10-dicarboxylic acid [3] **22B** (63 mg, 0.3 mmol) and PCl_5 (135 mg, 0.65 mmol) in dry benzene (2 mL) was stirred at 40–50 °C until all dissolved. After additional 15 min of stirring the solvent and $POCl_3$ were removed under reduced pressure. The resulting crude acid chloride was dissolved in dry CH_2Cl_2 (2 mL), 4-pentylphenol (105 mg, 0.64 mmol) was added followed by dry Et_3N (0.10 mL). The mixture was stirred at ambient temperature for 3 h, concentrated, and passed through a silica gel plug. The plug was washed with CH_2Cl_2 and the eluent was evaporated. The residue (165 mg) was recrystallized from isooctane, then MeCN, and finally from isooctane to give 95 mg (75% yield) of diester **18B** as colorless prisms: ^{1}H NMR (300 MHz) δ 0.91 (t, *J* = 6.1 Hz, 6H), 1.30–1.41 (m, 8H), 1.5–4.0 (brm, 8H), 1.65 (quint, *J* = 7.3 Hz, 4H), 2.65 (t, *J* = 7.7 Hz, 4H), 7.21 (d, *J* = 8.6 Hz, 4H), 7.27 (d, *J* = 8.6 Hz, 4H). Anal. Calcd for $C_{26}H_{38}B_8O_4$: C, 62.32; H, 7.64. Found: C, 62.42; H, 7.64.

Bis[4-(propoxycarbonyl)phenyl] 1,12-dicarba-*closo*-dodecaborane-1,12-dicarboxylate (**19A**).

A suspension of *p*-carborane-1,12-dicarboxylic acid **22A** (40 mg, 0.18 mmol) and PCl_5 (76 mg, 0.35 mmol) in $POCl_3$ (1 mL) was refluxing until all dissolved (1 h). After additional 15 min of stirring $POCl_3$ was removed under reduced pressure. The resulting crude acid chloride was dissolved in dry CH_2Cl_2 (2 mL), propyl 4-hydroxybenzoate (**23**, 66 mg, 0.37 mmol) was added followed by dry pyridine (0.03 mL). The mixture was stirred at ambient temperature overnight and washed with 5% HCl. Organic products were extracted (CH_2Cl_2), extracts dried (Na_2SO_4) and concentrated, and the residue passed through a silica gel plug. The plug was washed with CH_2Cl_2 and the eluent was evaporated to give 80 mg (82% yield) of a white solid which was repeatedly recrystallized from AcOEt/EtOH and isooctane/toluene mixture: ^{1}H NMR (300 MHz)

δ 1.02 (t, *J* = 7.4 Hz, 6H), 1.5–4.0 (brm, 10H), 1.78 (sex, *J* =7.1 Hz, 4H), 4.27 (t, *J* = 6.5 Hz, 4H), 7.06 (d, *J* = 8.7 Hz, 4H), 8.05 (d, *J* = 8.6 Hz, 4H). Anal. Calcd for $C_{24}H_{32}B_{10}O_8$: C, 51.79; H, 5.79. Found: C, 52.00; H, 5.72.

Bis[4-propoxycarbonyl)phenyl] 1,10-dicarba-*closo*-decaborane-1,10-dicarboxylate (**19B**).

It was prepared in 90% yield from **22B** and **23** according to the procedure described for ester **19A**. The white solid ester was repeatedly recrystallized from isooctane/toluene mixture and then MeCN: ^{1}H NMR (400 MHz) δ 1.05 (t, *J* =7.4 Hz, 6H), 1.5–4.0 (brm, 8H), 1.82 (sex, *J* =7.1 Hz, 4H), 4.32 (t, *J* = 6.7 Hz, 4H), 7.41 (d, *J* = 8.8 Hz, 4H), 8.18 (d, *J* = 8.8 Hz, 4H). Anal. Calcd for $C_{24}H_{30}B_8O_8$: C, 54.08; H, 5.67. Found: C, 53.93; H, 5.68.

Bis[4-(propoxycarbonyl)phenyl] bicyclo[2.2.2]octane-1,4-dicarboxylate (**19C**).

To the suspension of bicyclo[2.2.2]octane-1,4-dicarboxylic acid (**22C**, 60 mg, 0.3 mmol), propyl 4-hydroxybenzoate (**23**, 111 mg, 0.6 mmol) and PPh_3 (157 mg, 0.6 mg) in dry THF (2 ml), dimethyl azodicarboxylate (DMAD, 90 mg, 0.6 mmol) was added. The mixture was stirred at ambient temperature overnight, solvent was evaporated and the residue was passed through a silica gel plug (CH_2Cl_2) to give 50 mg (33% yield) of a white solid which was repeatedly recrystallized from EtOH and isooctane/toluene mixture: ^{1}H NMR (400 MHz) δ 1.03 (t, *J* = 7.4 Hz, 6H), 1.79 (sex, *J* =7.0 Hz, 4H), 2.07 (s, 12H), 4.28 (t, *J* = 6.7 Hz, 4H), 7.12 (d, *J* = 8.8 Hz, 4H), 8.08 (d, *J* = 8.8 Hz, 4H). Anal. Calcd for $C_{30}H_{34}O_8$: C, 68.95; H, 6.56. Found: C, 68.94; H, 6.56.

Bis[4-(butanoyloxyphenyl] 1,12-dicarba-*closo*-dodecaborane-1,12-dicarboxylate (**20A**).

It was prepared in 62% yield from **22A** and **24** according to the procedure described for ester **19A**. The white solid ester was repeatedly recrystallized from EtOH and then from isooctane: ^{1}H NMR (300 MHz) δ 1.03 (t, *J* = 7.4 Hz, 6H), 1.5–4.0 (brm, 10H), 1.76 (sex, *J* =7.4 Hz, 4H), 2.52 (t, *J* = 7.4 Hz, 4H), 6.99 (d, *J* = 9.0 Hz, 4H), 7.07 (d, *J* = 9.0 Hz, 4H). Anal. Calcd for $C_{24}H_{32}B_{10}O_8$: C, 51.79; H, 5.79. Found: C, 51.89; H, 5.78.

Bis[4-(butanoyloxy)phenyl] 1,10-dicarba-*closo*-decaborane-1,10-dicarboxylate (**20B**).

It was prepared in 65% yield from **22B** and **24** according to the procedure described for ester **19C**. The colorless ester was repeatedly recrystallized from hexane and then EtOH: ^{1}H NMR (400 MHz) δ 1.06 (t, *J* = 7.4 Hz, 6H), 1.5–4.0 (brm, 8H), 1.80 (sex, *J* =7.1 Hz, 4H), 2.57 (t, *J* = 7.4 Hz, 4H), 7.20 (d, *J* = 9.0 Hz, 4H), 7.34 (d, *J* = 9.0 Hz, 4H). Anal. Calcd for $C_{24}H_{30}B_8O_8$: C, 54.08; H, 5.67. Found: C, 54.34; H, 5.55.

Bis[4-(butanoyloxy)phenyl] bicyclo[2.2.2]octane-1,4-dicarboxylate (**20C**).

It was prepared in 62% yield from **22C** and **24** according to the procedure described for ester **19C**. The white solid ester was repeatedly recrystallized from EtOH and then isooctane: ^{1}H NMR (400 MHz) δ 1.04 (t, *J* = 7.4 Hz, 6H), 1.78 (sex, *J* =7.4 Hz, 4H), 2.04 (s, 12H), 2.53 (t, *J* = 7.4 Hz, 4H), 7.05 (d, *J* = 9.2 Hz, 4H), 7.09 (d, *J* = 9.2 Hz, 4H). Anal. Calcd for $C_{30}H_{34}O_8$: C, 68.95; H, 6.56. Found: C, 68.73; H, 6.50.

Bis[4-(butanoyloxy)phenyl] terephthalate (**20D**).

A suspension of terephthalic acid (**22D**, 200 mg, 1.2 mmol) and PCl_5 (500 mg, 2.50 mmol) in $POCl_3$ (2 mL) was refluxing until all dissolved (1 h) and all $POCl_3$ was removed under reduced pressure. The resulting crude terephthaloyl chloride was dissolved in dry CH_2Cl_2 (4 mL), 4-butanoyloxyphenol (**24**, 410 mg, 2.5 mmol) was added followed by dry pyridine (0.21 mL). The mixture was stirred at ambient temperature for 5 h and washed with 5% HCl. Organic products were extracted (CH_2Cl_2), extracts dried (Na_2SO_4) and concentrated and the residue passed through a silica gel plug. The plug was washed with CH_2Cl_2 and the eluent was evaporated to give 236 mg (40% yield) of a white solid which was repeatedly recrystallized from AcOEt/EtOH and isooctane/toluene mixtures: ^{1}H NMR (500 MHz) δ 1.06 (t, *J* = 7.4 Hz, 6H), 1.80 (sex, *J* = 7.4 Hz, 4H), 2.56 (t, *J* = 7.4 Hz, 4H), 7.17 (d, *J* = 7.1 Hz, 4H), 7.26 (d, *J* = 7.0 Hz, 4H), 8.33 (s, 4H). Anal. Calcd for $C_{28}H_{26}O_8$: C, 68.56; H, 5.34. Found: C, 68.54; H, 5.35.

1,12-Bis(4-hydroxyphenyl)-1,12-dicarba-*closo*-dodecaborane (**21A**) [4].

A 1M solution of BBr_3 in CH_2Cl_2 (5 mL, 5 mmol) was added dropwise to a solution of 1,12-bis(4-methoxyphenyl)-*p*-carborane [4] (**14A[0]**, 356 mg, 1 mmol) in dry CH_2Cl_2 (5 mL) at −78 °C. After stirring for 2 h at room temperature, the mixture was poured into ice and extracted with AcOEt. The organic layer was washed with brine, dried ($MgSO_4$), and then concentrated. The residue was purified by column chromatography on silica gel with AcOEt/*n*-hexane (gradient from 1:10 to 1:1) to give 317 mg (97% yield) of diol **21A** as a pale yellow powder: ^{1}H NMR (270 MHz), δ 1.50–3.70 (brm, 10H) 6.49 (d, *J* = 8.7 Hz, 4H), 6.94 (d, *J* = 8.7 Hz, 4H). HRMS, calcd. for $C_{14}H_{20}B_{10}O_2$: 328.2460. Found: 328.2446.

1,4-Bis(4-hydroxyphenyl)benzene (**21D**) [1].

To a suspension of 1,4-bis(4-methoxyphenyl)benzene (**14D[0]**, 580 mg, 2 mmol) in dry dichloroethane (10 mL) was added dropwise 1 M solution of BBr_3 in CH_2Cl_2 (6 mL, 6 mmol) at 0 °C. After stirring for 8 h at room temperature, the mixture was poured into ice and insoluble solid was collected and washed with water and CH_2Cl_2. The colorless solid was dissolved in THF, and purified by column chromatography on silica gel with 1:1 AcOEt:*n*-hexane to give 509 mg (97% yield) of diol **21D** as a colorless solid. Colorless prisms were obtained from AcOEt: mp >300 °C (lit. [1] mp 375 °C); ^{1}H NMR (270 MHz, DMSO) δ 6.84 (d, *J* = 8.7 Hz, 4H), 7.50 (d, *J* = 8.6 Hz, 4H), 7.60 (s, 4H), 9.52 (s, 2H); MS (EI) *m/z* 262 (M^+, 100%). Anal. Calcd for $C_{18}H_{14}O_2$: C, 82.42; H, 5.38; Calcd for $C_{18}H_{14}O_2$•1/4H_2O: C, 81.03; H, 5.48. Found: C, 81.05; H, 5.49.

4-(Butanoyloxy)phenol (**24**) [5].

To the solution of *p*-(benzyloxy)phenol (6.0 g, 30 mmol) in dry CH_2Cl_2 (20 ml) butyryl chloride (3.2 g, 30 mmol) was added followed by dry pyridine (2.5 g, 31 mmol). The mixture was stirred for 4 h at ambient temperature, washed with 5% of HCl, extracted (CH_2Cl_2), dried (Na_2SO_4), evaporated and purified on silica gel plug (CH_2Cl_2/hexane, 1:2) to give 6.2 g (75% yield) of *p*-(benzyloxy)phenyl butyrate as a white solid: lit [5] mp 79–82 °C; ^{1}H NMR (400 MHz) δ 1.04 (t, *J* =7.4 Hz, 3H), 1.77 (sex, *J* =7.4 Hz, 2H), 2.52 (t, *J* = 7.4 Hz, 2H), 5.05 (s, 2H), 6.96 and 6.99 (pseudo AB, *J* = 9.4 Hz, 4H), 7.31–7.44 (m, 5H).

Without further purification, the product was dissolved in THF (20 ml), Pd/C (240 mg, 0.23 mmol, 10%) was added, and the mixture was kept under an atmosphere of hydrogen overnight.

The solvent was evaporated and the residue was purified on a silica gel funnel (CH_2Cl_2) to give 3.3 g (80% yield) of white solid: mp 50–52 °C (lit. [5] mp 53–55 °C); ^{1}H NMR (300 MHz) δ 1.03 (t, *J* =7.4 Hz, 3H), 1.77 (sex, *J* =7.4 Hz, 2H), 2.52 (t, *J* = 7.4 Hz, 2H), 4.82 (s, 1H), 6.78 (d, *J* = 8.8 Hz, 2H), 6.92 (d, *J* = 8.8 Hz, 2H).

3. Thermal Analysis

Table 1: Transition temperatures (°C) and enthalpies (kJ/mol) for selected liquid crystals.[a]

	*	A	B	C	D
		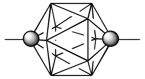	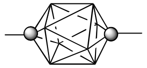	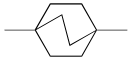	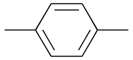
	X	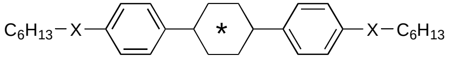			
**14[6]**	-CH_2O-(Ph)	Cr 96 N 98 I[b]	Cr 73 N 105 I[b]	Cr 98 SmB 161 SmA 179 I[b]	Cr_1 108 Cr_2 182 SmF 218 SmI 219 SmC 232 SmA 235 I[b]
**15[6]**	-OOC-(Ph)	Cr 112 (N 31) I[b]	Cr 65 (N 11) I[b]	Cr_1 100 Cr_2 114 SmA 148 I[b]	Cr 134 SmC 143 SmA183 I[b]
**16[6]**	-COO-(Ph)	Cr 108 N 132 I<br>(34.3) (2.4)	Cr_1 73 Cr_2 102 N 136 I<br>(14.9) (25.4) (2.0)	Cr_1 33 Cr_2 102 X 205 N 207 I<br>(11.6) (18.5) (10.6)[d]	Cr 66 X 96 SmF 226 SmI 232 SmC[c] 250 SmA 251 I<br>(3.2) (1.9) (3.7) (6.7) (13.4)[d]
		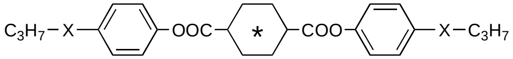			
**17**	-CH_2O-(Ph)	Cr 137 N 182.6 I[e]	Cr_1 70 Cr_2 111 N 183.4 I[e]	Cr 112 N 229.5 I[e]	Cr 189 N 235 I[f]
**18**	-CH_2CH_2-(Ph)	Cr 106 N 118 I [g]<br>(31.6) (1.4)	Cr 85 N 110 I<br>(27.3) (1.6)	Cr 98 N 173 I[h]	Cr 155 N 181 I[i]
**19**	-OOC-(Ph)	Cr 203 (N 139)[c] I<br>(69.5)	Cr 160 (N 128) I<br>(52.7) (0.4)	Cr 121 N 195 I<br>(48.8) (0.2)	Cr 130 SmA 207 N 221 I[j]
**20**	-COO-(Ph)	Cr 133 N 230 I<br>(39.2) (2.0)	Cr 120 N 234 I<br>(15.8) (1.0)	Cr 133 N 275 I<br>(30.8) (1.3)	Cr 230 N 287 I<br>(56.9) (1.5)

[a]Obtained on heating; Cr: crystal, S: smectic, N: nematic, I: isotropic, X: unidentified phase.
[b]Ref. [2]
[c]Optical determination obtained on cooling.
[d]Combined enthalpies for two transitions.
[e]Ref [6]
[f]Ref. [7]
[g]Previously reported Cr 104 N 114 I, ref. [8]
[h]Ref. [9]
[i]Ref. [10]
[j]Ref [11]

Table 2: Transition temperatures (°C) and enthalpies (kJ/mol) for **16A[n]**.[a]

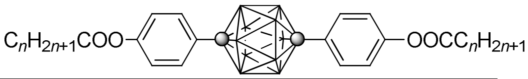

n	Transition temperatures
5	Cr_1 66 Cr_2 120 N 155 I<br>(15.5) (26.9) (2.4)
6	Cr 108 N 132 I<br>(34.3) (2.4)
7	Cr_1 76 Cr_2 92 N 124 I<br>(30.6) (27.8) (1.9)

[a]Obtained on heating; Cr: crystal, N: nematic, I: isotropic.

4. References

1. Price, C. C.; Mueller, G. P. *J. Am. Chem. Soc.* **1944,** *66,* 632–634. doi:10.1021/ja01232a038
2. Kaszynski, P.; Kulikiewicz, K. K.; Januszko, A.; Douglass, A. G.; Tilford, R. W.; Pakhomov, S.; Patel, M. K.; Ke, Y.; Radziszewski, G. J.; Young, V. G., Jr. submitted.
3. Garrett, P. M.; Smart, J. C.; Hawthorne, M. F. *J. Am. Chem. Soc.* **1969,** *91,* 4707–4709. doi:10.1021/ja01045a021
4. Fox, M. A.; MacBride, J. A. H.; Peace, R. J.; Wade, K. *J. Chem. Soc., Dalton Trans.* **1998,** 401–412. doi:10.1039/a707154j
5. Neubert, M. E.; Wildman, P. J.; Zawaski, M. J.; Hanlon, C. A.; Benyo, T. L.; De Vries, A. *Mol. Cryst. Liq. Cryst.* **1987,** *145,* 111–158. doi:10.1080/00268948708080217
6. Kaszynski, P.; Januszko, A.; Ohta, K.; Nagamine, T.; Potaczek, P.; Young, V. G., Jr.; Endo, Y. *Liq. Cryst.* **2008,** *35,* 1169–1190. doi:10.1080/02678290802409775
7. Kelker, H.; Scheurle, B. *J. Phys. (Paris)* **1969**, *30-C4,* 104–108. doi: 10.1051/jphyscol:1969425
8. Kaszynski, P.; Huang, J.; Jenkins, G. S.; Bairamov, K. A.; Lipiak, D. *Mol. Cryst. Liq. Cryst.* **1995,** *260,* 315–332. doi:10.1080/10587259508038705
9. Compound ID # 37494 in LiqCryst 4.6 database.
10. Neubert, M. E.; Stahl, M. E.; Cline, R. E. *Mol. Cryst. Liq. Cryst.* **1982,** *89,* 93–117. doi:10.1080/00268948208074472
11. Leblanc, J. P.; Tessier, M.; Judas, D.; Friedrich, C.; Noël, C.; Maréchal, E. *Macromolecules* **1993,** *26,* 4391–4399. doi:10.1021/ma00069a001
